# Maternal Photoperiodic Programming: Melatonin and Seasonal Synchronization Before Birth

**DOI:** 10.3389/fendo.2019.00901

**Published:** 2020-01-10

**Authors:** Jayme van Dalum, Vebjørn J. Melum, Shona H. Wood, David G. Hazlerigg

**Affiliations:** Department of Arctic and Marine Biology, UiT – the Arctic University of Norway, Tromsø, Norway

**Keywords:** melatonin, pars tuberalis, tanycyte, fetal programming, thyrotropin (TSH—thyroid-stimulating hormone), photoperiodic history, deiodinase, thyroid hormone (T3)

## Abstract

This mini-review considers the phenomenon of maternal photoperiodic programming (MPP). In order to match neonatal development to environmental conditions at the time of birth, mammals use melatonin produced by the maternal pineal gland as a transplacental signal representing ambient photoperiod. Melatonin acts via receptors in the fetal pituitary gland, exerting actions on the developing medio-basal hypothalamus. Within this structure, a central role for specialized ependymal cells known as tanycytes has emerged, linking melatonin to control of hypothalamic thyroid metabolism and in turn to pup development. This review summarizes current knowledge of this programming mechanism, and its relevance in an eco-evolutionary context. Maternal photoperiodic programming emerges as a useful paradigm for understanding how *in utero* programing of hypothalamic function leads to life-long effects on growth, reproduction, health and disease in mammals, including humans.

## Introduction

Life on a rotating planet brings predictable daily and seasonal environmental challenges to the balancing of energy budgets for biological fitness. Because thermo-energetic challenges are inversely related to body size, the capacity to predict the cyclical environmental changes is of special importance for small animals (1), presumably this is crucial in the neonatal/juvenile period. The light-dark cycle and annually changing day lengths (photoperiod), are the most predictable information sources regarding the time of the day and time of the year. Adult mammals are in direct contact with the photic environment, and translate this signal via the hormone melatonin, to time their own changes in physiology and behavior. Contrastingly, the fetus is isolated from photoperiodic information both because light levels *in utero* are much lower than in the surrounding environment, and light sensing pathways are not fully developed until after birth in many cases (2, 3). To deal with this challenge, mammals use maternal melatonin as a transplacental signal (4), through which the fetus gains information about time of day [for review see (5, 6), and references therein], and about time of year [for review see (7), and references therein]. Several articles in this review series deal with the former aspect, and so we focus here on the latter, which we describe as maternal photoperiodic programming (MPP). We first discuss the eco-evolutionary importance of MPP; then we go on to review current understanding of how MPP takes place, focussing on the sites of action of melatonin during the fetal and neonatal period.

## The Evolutionary Drivers For MPP

**Figure 1:**
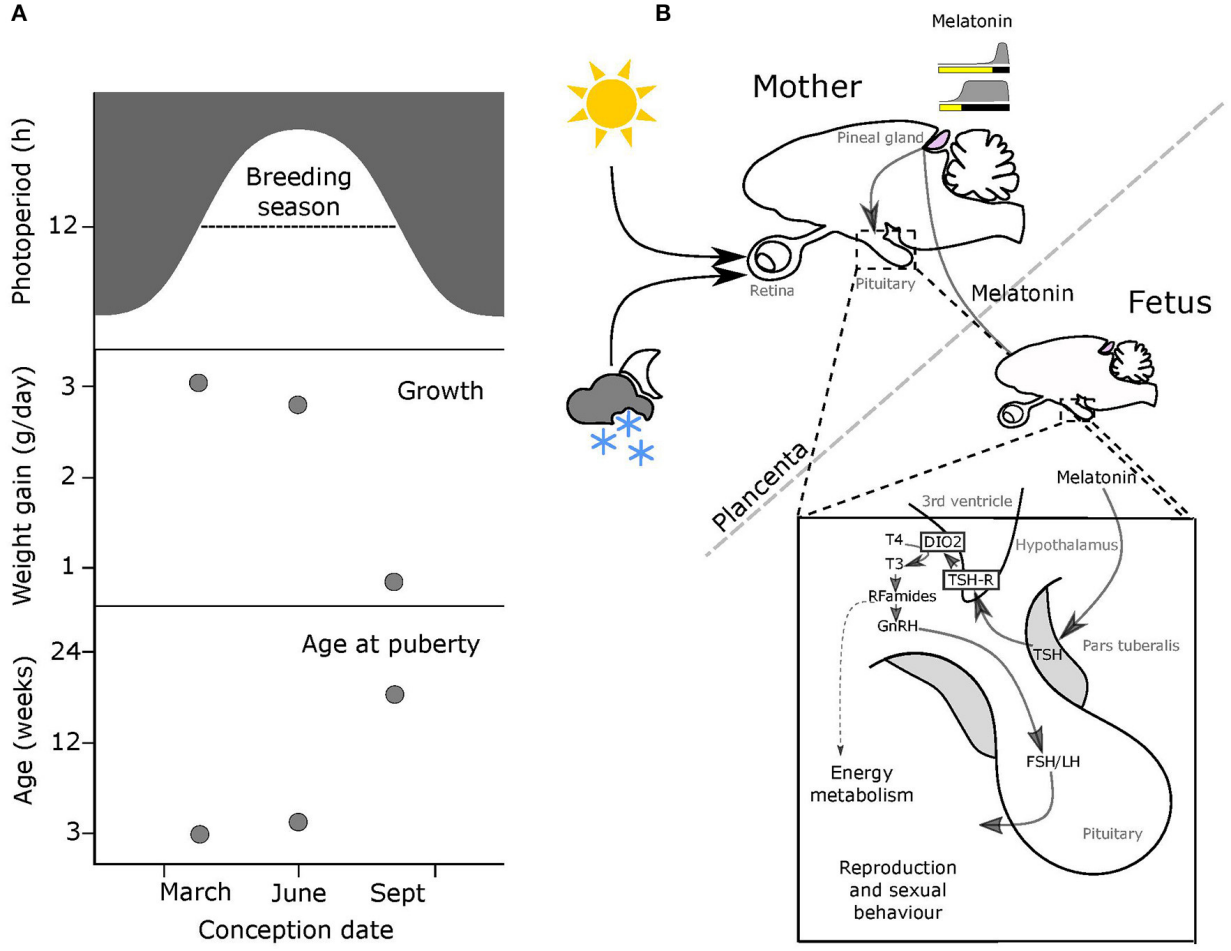
Melatonin-mediated transplacental relay of photoperiodic information. **(A)** The breeding season for small rodents runs from spring through to early autumn (top panel, dashed line). Middle & bottom panels: offspring born early in the breeding season on increasing photoperiods grow fast and breed in the same season, while pups born later on declining photoperiods grow slowly and delay breeding to the following year. **(B)** Actions of maternal melatonin via the pars tuberalis (PT). In both the mother and the fetus, thyrotrophs in the *pars tuberalis* (PT) contain melatonin receptors (MT1), and in response to shorter melatonin signals representing intermediate to long photoperiods these cells secrete thyroid stimulating hormone (TSH). Tanycytes lining the 3rd ventricle, express TSH receptors, and respond to changing levels of PT TSH secretion by modulating relative levels of expression of two thyroid hormone deiodinase enzymes (dio2 and dio3). This affects the local thyroid environment in the MBH, with relatively increased dio2 expresison causing a relative increase in levels of T3 (the active form of TH). This in turn determines the reproductive behavior and energy metabolism of the adult animal.

While seasonal conditions at any given point in the annual cycle may vary considerably from year to year, photoperiod is the most reliable cue for position in the annual cycle, and hence is a predictor of forthcoming environmental challenges. This in essence is the ultimate evolutionary reason for the evolution of melatonin-based photoperiodic synchronization in mammals. It is also important to appreciate that absolute day length alone is insufficient as a synchronizing signal because all variations in day length, except the solsticial maxima and minima, occur twice in every solar year. Hence the use of photoperiod as a cue must be dependent on prior history of photoperiodic exposure: intermediate photoperiods preceded by the long days of summer presage autumn and winter, whereas intermediate photoperiods preceded by the short days of winter presage spring and summer (Figure 1A) [for review see (8)].

This importance of integrating photoperiodic history into the use of photoperiod as a cue is made abundantly clear by a consideration of reproductive development and life-history strategy in short lived rodent species including voles and hamsters (9–11). In such animals the time from conception to reproductive maturity is potentially <2 months, and so multiple generations are typically born within a single annual breeding season. Nonetheless, the optimal life-history strategy for individuals born in the spring is entirely different from that for individuals born late in the breeding season (Figure 1A). For the former a “live fast, die young” strategy with fitness success based on producing progeny within the same summer season is appropriate because within the same season there will continue to be sufficient resources for lactation and rearing young. Contrastingly, young born later in the season do not have time for breeding and rearing of young before the autumn decline in resources and increased thermo-energetic demand occurs. As consequence these late born pups delay reproduction until the following year, conserving resources for investment in overwintering survival. In the field, the use of these two alternate life-history strategies as a function of time of birth reveals itself as a bimodal age distribution in wild caught individuals (9–11).

## Characterization of MPP in the Laboratory

In the laboratory it is possible to reveal these alternate strategies simply by manipulation of artificial photoperiod. In the Montane vole (*Microtus montanus*), pups gestated and raised under long photoperiods (16L:8D) delay growth and maturation when exposed to shorter, intermediate photoperiods (14L:10D) at weaning, whereas pups gestated under short photoperiods (8L:16D) undergo accelerated growth and maturation when exposed to the same intermediate photoperiod (12, 13). The use of intermediate photoperiods is a powerful paradigm to show that weaned offspring have a “memory” of prior photoperiodic history. Determining if this “memory” is encoded *in utero* or neonatally, was a challenge addressed by a series of elegant studies by Milton Stetson, Teresa Horton and colleagues, which dissected the origins of this photoperiodic history, both through cross-fostering experiments and by resolving photoperiodic manipulation into gestational, neonatal and post-weaning phases [(12, 14, 15), reviewed in (7, 16)].

Cross fostering experiments in Montane voles demonstrate that the *in utero* environment is where the programming of developmental trajectories occurs (14). Pregnant mothers were kept under long (16L:8D) or short (8L:16D) photoperiods. At birth, half of the young were given to a foster mother who had experienced the same photoperiod as the birth-mother and the other half of the young went to a foster mother who had experienced the opposite photoperiod during pregnancy, compared to the birth-mother. All young were raised under intermediate (14L:10D) photoperiods after birth. The accelerated growth and sexual maturation of short-day gestated voles compared to long-day gestated voles clearly demonstrated the *in utero* transfer of photoperiodic information by the actual birth-mother. The foster mother's photoperiodic history had no effect on the offspring after birth, which excludes the effect of maternal signals transferred through milk. Similar effects of maternal photoperiodic programming have been shown in Siberian hamsters (*Phodopus sungorus*) (15, 17), collared lemmings (*Dicrostonyx groenlandicus*) (18), and meadow voles (*Microtus pennsylvanicus*) (19, 20).

The clear conclusion from these studies is that photoperiod influences reproductive development in a manner dependent on the interaction between photoperiod exposure *in utero* and photoperiod exposure post-weaning. Photoperiod exposure in the intervening neonatal period has little influence, and constitutes a “dead zone” for MPP, probably because at this stage the photo-neuroendocrine system (PNS) is not fully light- responsive and pups typically remain in subterranean nests (21).

## MPP in Non-Rodent Species

Longer-lived, larger mammals also show evidence of MPP. Sexual maturity of red deer gestated under short photoperiods is advanced compared to long photoperiods (22). The effect of gestation is also evident in the prolactin levels of sheep lambs at the time of birth, with levels being lower in short-day gestated lambs than in long-day gestated lambs (23). Moreover, subsequent responses to intermediate (LD12:12) photoperiods after birth were quite different, with prolactin levels rapidly increased in short-day gestated lambs but decreased in long-day gestated lambs. Under natural conditions sheep and other ungulates have a single round of reproduction in a given year, and this is tightly constrained to an autumn period to ensure that young are born in the spring. Hence in contrast to voles and hamsters, an evolutionary narrative based on alternate life-history strategies cannot apply. Rather it is likely that *in utero* programming establishes the phase for calendar timer mechanisms from birth which then continue throughout life.

## Role of Melatonin in MPP

Except in early development, the pineal gland of mammals secretes melatonin in a light responsive fashion. The photic input pathway from the retina to the suprachiasmatic nucleus (SCN) drives rhythmic melatonin production from the pineal gland and this melatonin signal is sculpted by photoperiod to provide an internal endocrine representation for external photoperiod, this is the PNS (Figure 1B) [for review see (8, 24)]. Through this means, short (winter) photoperiods are represented by increased duration of nocturnally elevated plasma melatonin titers and long (summer) photoperiods by shorter duration for nocturnally elevated titers (Figure 1B).

The pivotal role of maternal pineal melatonin production in MPP was first demonstrated by a series of studies in Siberian hamsters (*P. sungorus*) [(17, 25–27), for review see (28)]. Injection of melatonin to pineal-intact mothers caused a suppression of pup testicular growth, dependent on the phase of melatonin injection relative to the light dark cycle. Specifically, injections in afternoon were most effective, because melatonin delivered at this phase extended the endogenous maternal melatonin signal to give it a profile mimicking a short photoperiod (25). Complete removal of the maternal melatonin signal by pinealectomy (px) blocked the effect of *in utero* photoperiod manipulations on pup development (26), as did fitting of pineal-intact mothers with continuous release melatonin implants (27). Collectively, these studies reveal that maternal pineal melatonin production relays information about ambient photoperiod to the developing fetus.

## Melatonin Sites of Action in the Developing Fetus

The use of the radio-analog of melatonin, 2-iodo-melatonin (29), led to the identification of melatonin binding sites in a range of central and peripheral fetal tissues (30). In fetal rodents, melatonin binding sites representing high affinity G–protein coupled receptors are consistently observed in the pars tuberalis (PT) and pars distalis (PD) of the pituitary and in the SCN [(31–33), for review see (30, 34)]. While type 1 melatonin receptor (mt1) expression disappears from the PD within a few days of birth (35), expression in the PT persists, and this site has emerged as the key site for the seasonal actions of melatonin in adult mammals [for review see (8, 30, 36–38)].

The PT shows the highest concentration of melatonin receptors of all mammalian tissues, and these mediate photoperiodic control of TSH production by the PT through a circadian-based “coincidence timer” mechanism (39, 40). TSH produced by the PT acts locally on the TSH receptors (TSHR) expressed in tanycyte cells lining the third ventricle of the hypothalamus (41, 42). Ligand binding to TSHR regulates the expression of deiodinase seleno-enzymes (Dio2 and Dio3), which in turn controls the local metabolism of thyroid hormone within the mediobasal hypothalamus (MBH), driving seasonal adaptations (Figure 1B) [(41, 42), for review see (24, 43)].

## Fetal PT as a Target for the Maternal Melatonin Signal

Based on the paradigm emerging in adult mammals, Sáenz de Miera and colleagues have explored the involvement of the PT and MBH in MPP (44). This study demonstrates that in the Siberian hamster, expression of *TSH* in the fetal PT at the time of birth depends on maternal photoperiod, with high expression in pups gestated on LP but low expression in pups gestated on SP. These effects on PT *TSH* gene expression persisted through the perinatal period. As in adult mammals, *TSHR* expression is found in the ependymal region, and corresponding effects of photoperiod on the expression of dio2 and dio3 were observed (i.e., high *dio2* and low *dio3* in LP gestated pups and the converse in SP gestated pups). These studies provide evidence that the fetal PT mediates seasonal programming effects of maternal melatonin.

## MPP Establishes Photoperiodic History-Dependence at the Level of the Tanycytes

**Figure 2:**
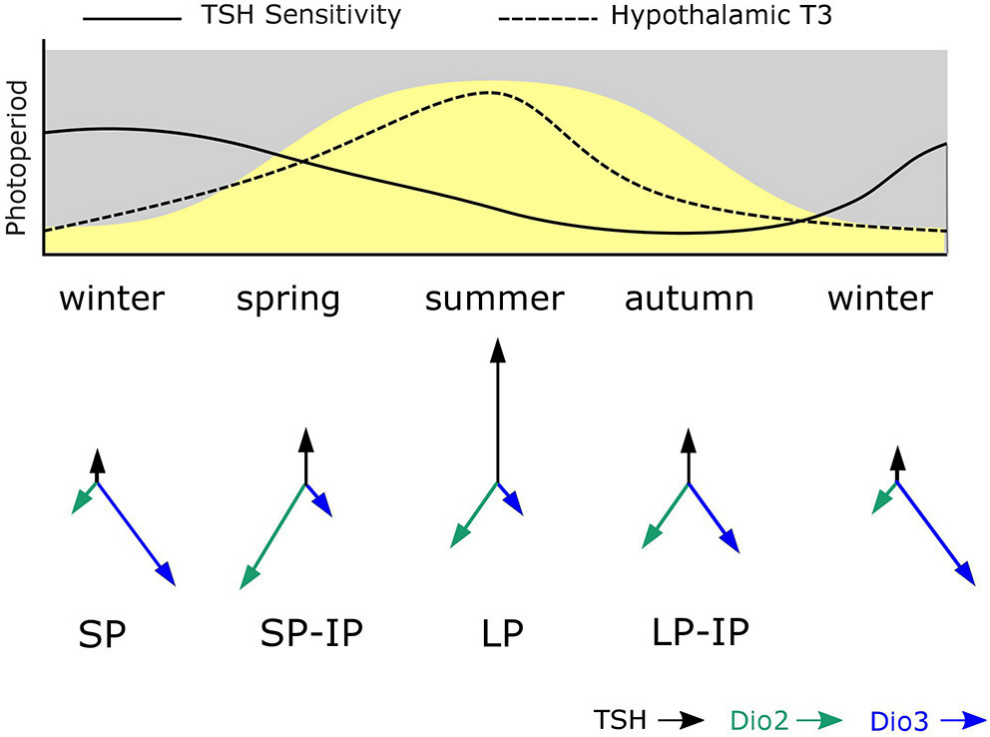
Model for photoperiodic history-dependence through shifting tanycyte sensitivity to TSH. The solid line in the upper panel shows how tanycyte sensitivity to TSH is presumed to change during the course of the year. During winter PT TSH secretion is photoperiodically inhibited and TSH-sensitivity becomes heightened. In spring increased TSH production is a potent stimulus for increased dio2 expression due to high TSH sensitivity established in the winter phase. As spring becomes summer, sensitivity to TSH in the tanycytes declines and so high dio2 expression is not maintained despite continued exposure to long photoperiods. Then in autumn, the combination of declining TSH secretion and reduced sensitivity to TSH established in the summer phase leads to loss of dio2 expression and increased dio3 expression. The system then resets to the winter. The predicted consequences of this for hypothalamic T3 levels is shown as a dashed line—the asymmetry of this relative to the curve for photoperiod represents photoperiodic history-dependence. The lower panel shows the predicted consequences of this process for TSH, dio2 and dio3 expression—where arrow lengths represent strength of expression.

Maternal photoperiod not only sets neonatal levels of TSH and deiodinase gene expression, associated with different trajectories for gonadal development, it also influences the sensitivity of MBH deiodinase gene expression to photoperiod exposure post-weaning. Specifically SP-gestation was associated with more dio2 and less dio3 expression in response to intermediate photoperiods than was the case for LP-gestated pups (Figure 2). Hence MPP is seen in hypothalamic expression of the key enzymes controlling thyroid status in the developing hypothalamus.

This effect does not seem to derive from downstream programming of both melatonin synthesis in the weaned pups, and sensitivity to melatonin at the level of the pup PT, but rather, it derives from history-dependent differences in sensitivity to TSH produced by the pup PT. This was demonstrated by icv injection of exogenous TSH which had a bigger inductive effect on *dio2* expression in SP- than in LP-gestated pups (Figure 2). Since no overt changes in *TSHR* expression in the MBH were seen in these experiments (44), other causes for this apparent shift in TSH sensitivity must be sought.

The identification of tanycytes as the site at which MPP generates photoperiodic history dependence echoes data from studies in the Soay sheep (45). Here, the onset of refractoriness to SP-exposure, i.e., another example of photoperiodic history-dependence, also appears at the level of *dio2/dio3* expression in tanycytes independently of changes in *TSH* expression in the PT.

## Functional Roles for Hypothalamic Tanycytes

If the significance of these programming phenomena are to be properly understood it is imperative that attention focuses on tanycyte function. Tanycytes are a specialized form of ependymal cell derived from a glial cell lineage shared with microglial cells—for review see (46–49). They differ morphologically from the cuboidal epithelial cells that line most of the ventricular walls in that they have a bipolar morphology with extensive processes projecting into the parenchymal tissue surrounding the ependymal zone. Detailed analysis suggests that hypothalamic tanycytes may be subclassified based upon their anatomical location and upon their expression profiles (50)—but how these differences relate to differences in function remains uncertain. Much has been written on the possible functions of these cells, and at least three broad classes of cellular process have emerged: metabolic sensing (48, 51–53) regulation of blood/CSF/brain interfaces (50) and neurogenesis (54). The regulation of deiodinase gene expression and consequent effects on the local thyroid environment is but one molecular function of tanycytes, and may impact on any or all of the above cellular processes. At one level dio2/dio3 are regulators of uptake of active thyroid hormone into the circumventricular environment, and so serve a role as enzymatic “gatekeepers” (55). At another level, because T3 levels in the hypothalamus interact with the AMP-kinase dependent energy sensing pathways (56), shifts in deiodinase expression may be linked to metabolic sensing and responses. Thirdly, because T3 is strongly implicated in neurogenic pathways (57–59), shifts in T3 status dependent on photoperiodic history may impact in neurogenesis-dependent neural plasticity in the basal hypothalamus (54, 60, 61). Much remains to be done to establish an integrated view on the consequences of photoperiodic programming of tanycyte function.

## MPP in the Wider Context of Programming by Early Life Experience

The life-long consequences of early life experience is a topic of major biomedical importance. Epidemiological studies in humans demonstrate a positive correlation between low birthweight and susceptibility to obesity and cardiovascular health problems in adult life (62–66). Attempts to understand the mechanisms behind this phenomenon have led to studies in rats, in which maternal undernutrition leads to a chronic increase in susceptibility to weight gain when fed a “cafeteria” diet (67). Remarkably, this effect is completely reversed by treatment with the lipostatic hormone, leptin, in a narrow window in the neonatal period, which has closed by 10 days post-partum [(67), for review see (68)]. The mechanisms behind this effect of leptin remain unclear, it is probably not a coincidence that the ependymal zone of the MBH expresses high levels of leptin receptor at post-natal day 4, which then decline rapidly over the following week (69). This pattern is the inverse of that seen in the arcuate nuclei, and points to a transient role for leptin in establishing energy regulatory circuits in the neonatal period. The mapping of leptin receptor expression to the region encompassing the tanycytes involved in MPP suggests that this region is at the crux of mechanisms through which hypothalamic control circuits are established in early life. For this reason, we suggest that MPP, which relies on a harmless and non-invasive environmental perturbation (i.e., light) and acts through a well-defined pharmacological pathway (i.e., MT1 receptors in the PT), is a useful experimental paradigm for investigating the mechanisms through which early life experience establishes long term patterns of hypothalamic regulation.

## Author Contributions

All authors listed have made a substantial, direct and intellectual contribution to the work, and approved it for publication.

### Conflict of Interest

The authors declare that the research was conducted in the absence of any commercial or financial relationships that could be construed as a potential conflict of interest.

## Abbreviations

Dio2: type 2 deiodinase
Dio3: type 3 deiodinase
MBH: medio basal hypothalamus
MPP: maternal photoperiodic programing
MT1: type 1 melatonin receptor
3V: 3rd ventricle
PNS: photoneuroendocrine system
PD: pars distalis
PT: pars tuberalis
Px: pinealectomy
SCN: suprachiasmatic nucleus
SCG: superior cervical ganglion
T3: triiodothyronine
T4: thyroxine
TSH: thyroid stimulating hormone.
